# Mechanical Stress Modulates Calcium-Activated-Chloride Currents in Differentiating Lens Cells

**DOI:** 10.3389/fphys.2022.814651

**Published:** 2022-01-31

**Authors:** Lisa Ebihara, Pooja Acharya, Jun-Jie Tong

**Affiliations:** ^1^Center of Proteomics and Molecular Therapeutics, Rosalind Franklin University of Medicine and Science, North Chicago, IL, United States; ^2^Discipline of Physiology and Biophysics, Rosalind Franklin University of Medicine and Science, North Chicago, IL, United States

**Keywords:** mechanosensitive channel, TMEM16A, anoctamin-1, chloride, lens, accommodation

## Abstract

During accommodation, the lens changes focus by altering its shape following contraction and relaxation of the ciliary muscle. At the cellular level, these changes in shape may be accompanied by fluid flow in and out of individual lens cells. We tested the hypothesis that some of this flow might be directly modulated by pressure-activated channels. In particular, we used the whole cell patch clamp technique to test whether calcium-activated-chloride channels (CaCCs) expressed in differentiating lens cells are activated by mechanical stimulation. Our results show that mechanical stress, produced by focally perfusing the lens cell at a constant rate, caused a significant increase in a chloride current that could be fully reversed by stopping perfusion. The time course of activation and recovery from activation of the flow-induced current occurred rapidly over a time frame similar to that of accommodation. The flow-induced current could be inhibited by the TMEM16A specific CaCC blocker, Ani9, suggesting that the affected current was predominantly due to TMEM16A chloride channels. The mechanism of action of mechanical stress did not appear to involve calcium influx through other mechanosensitive ion channels since removal of calcium from the bath solution failed to block the flow-induced chloride current. In conclusion, our results suggest that CaCCs in the lens can be rapidly and reversibly modulated by mechanical stress, consistent with their participation in regulation of volume in this organ.

## Introduction

The lens is an avascular, transparent organ whose primary function is to focus light on the retina. To compensate for the absence of blood vessels, the lens has developed an unusual type of circulation system that consists of circulating ionic fluxes that flow into the lens at the anterior and posterior pole and out of the lens at the equator (see Mathias et al., 2010 for review). These ionic fluxes, carried mainly by sodium, are associated with the flow of fluid into and through the lens which facilitates the delivery of nutrients to the inner fiber cells and the removal of metabolic wastes. They also play a critical role in volume regulation. Essential components of this lens circulation system include gap junctions, Na-K ATPases, aquaporins, and other channels and transporters such as TRPV4 and TRPV1 which have recently been shown to act as mechanosensors and modulate the flow of fluid through the lens in response to changes in intracellular hydrostatic pressure (Shahidullah et al., 2012b; Gao et al., 2015; Delamere et al., 2020) or mechanical loading exerted by the ciliary zonules on the lens (Chen et al., 2019).

Chloride channels and transporters are also thought to play an important role in volume regulation in the lens (see Donaldson et al., 2009 for review). According to a lens circulation model first proposed by Mathias et al. (1997), the driving force for chloride is outward at the periphery of the lens and changes to inward in the deeper layers of the lens. Since all of the cells in the lens are electrically coupled by gap junctions, this would be predicted to result in a circulating chloride current that could regulate the volume of the entire lens. Indeed, it has been shown experimentally that exposure of the lens to the non-selective chloride channel blocker, NPPB, under isotonic conditions, results in two distinct zones of tissue damage in the outer cortex: a peripheral zone of fiber cell swelling due to block of chloride efflux and a deeper zone of extracellular dilatation due to block of chloride influx (Tunstall et al., 1999; Young et al., 2000; Merriman-Smith et al., 2002; Chee et al., 2006).

Patch clamp studies, performed prior to the identification of the TMEM16 family of calcium-activated-channels, reported that peripheral fiber cells from the zone of chloride influx exhibited a constitutionally active, outwardly rectifying chloride current with slow, voltage-dependent activation kinetics, and a low field strength selectivity series (I^–^ > Cl^–^ > gluconate) (Webb et al., 2004; Webb and Donaldson, 2008, 2009). The anion selectivity and voltage gating properties of this current resembled those of classical calcium-activated chloride currents. In shorter fiber cells from the zone of chloride efflux, this chloride current was generally quiescent but could be activated by hypotonic challenge and/or addition of the KCC blocker, DIOA. More recently, we have shown that calcium-activated-chloride channels (CaCCs) composed of TMEM16A and TMEM16B were expressed in newly differentiating lens epithelial and fiber cells in wild-type and double knockout (KO) that lack both Cx50 and Cx46 using a combination of reverse transcript PCR (RT-PCR), immunohistochemistry and whole cell patch clamp techniques (Tong et al., 2019).

In the present study, we used the whole cell patch clamp technique to test the hypothesis that these CaCCs are mechanosensitive in lens cells isolated from double KO mouse lenses. One of the main advantages of using this preparation to perform electrophysiological experiments is that the isolation of fiber cells from lens of these KO mice can be performed using milder dissociation conditions than those used in past attempts to dissociate fiber cells, possibly due to alterations in cell adhesion properties (Wang et al., 2016; Hu et al., 2017). Another advantage is that the double KO fiber cells lack large, calcium-sensitive connexin hemichannel currents and can tolerate exposure to calcium-containing solutions without requiring the addition of non-specific cation channel blockers such as 1 mM Gadolinium to prevent fiber cell vesiculation and death (Ebihara et al., 2011; Tong et al., 2019). Our results demonstrate that CaCCs in dissociated lens can be rapidly and reversibly modulated by mechanical stress, consistent with their participation in regulation of volume in this organ.

## Materials and Methods

### Chemicals

Ani9 and CaCCinh-A01 were obtained from Tocris Bioscience (Bristol, United Kingdom). GSK1016790A was obtained from Sigma-Aldrich Chemicals Company (ST. Louis, MO, United States). All other chemicals were purchased from Sigma-Aldrich Chemicals Company (St. Louis, MO, United States) or Thermo Fisher Scientific (Waltham, MA, United States) unless otherwise specified.

### Mice

Transgenic Cx46(−/−) Cx50(−/−) double KO mice were generated as previously described (Ebihara et al., 2014). The transgenic mice were in a C57 genetic background. For tissue harvesting, 4–8-week-old mice were euthanized and eyes were extracted using procedures approved by the Rosalind Franklin University Animal Care and Use Committee.

### Dissociation of Differentiating Lens Cells

Lens cells were dissociated from double KO mouse lenses as previously described (Tong et al., 2019). Briefly, the capsule was removed from double KO mouse lenses and incubated in dissociation buffer (DB) containing 0.0625% collagenase (type IV; Worthington Biochemical, Lakewood, NJ, United States) and 0.025% protease (type XXIV; Sigma-Aldrich, St. Louis, MO, United States) at room temperature for 15 min. The capsule was washed once with DB and resuspended in DB. The epithelial and young fiber cells were then mechanically removed from the capsule by gentle trituration with a Pasteur pipette, pelleted (1,000 rpm for 2 min) and resuspended in DB. The isolated cells were plated out on the bottom of a plastic tissue culture dish. Once the cells attached to the bottom of the dish (∼10 min), they were overlaid with standard bath solution and used immediately for patch-clamp experiments.

### Patch Clamp Experiments

Membrane currents were recorded from isolated epithelial cells and newly differentiating fiber cells using the whole cell patch clamp technique. The cells ranged from 50 to 300 μm and had membrane capacitances measuring between 25 and 100 pF. All of the cells contained nuclei indicating that they were isolated from the outer region of the cortex near the equator (i.e., zone of fluid efflux of the lens). A 60 mm tissue culture dish was used as the recording chamber. A pinch valve-controlled, gravity driven perfusion system connected by thin tubing to a 500 μm ID millimanifold solution applicator (ALA Scientific, Farmingdale, NY, United States) was used to provide focal fluid flow at a rate ranging between 1.0 and 1.3 ml/min. The applicator was positioned ∼1 mm from the cell of interest using a micromanipulator. Only those cells that remained firmly attached to the bottom of the dish throughout the course of the experiment were used to investigate the effects of mechanical stress. We chose to use fluid flow as a method to activate mechanosensitive chloride channels primarily because of the high degree repeatability of its effects. The bath was grounded *via* a 1 mm diameter Ag/AgCl wire electrode mounted in a pipette tip filled with 3M KCl agar.

Currents were recorded using an Axoclamp 200B patch clamp amplifier (Molecular Devices, San Jose, CA, United States) or MultiClamp 700A patch clamp amplifier (Molecular Devices, San Jose, CA, United States), filtered at 1 kHz with a low-pass filter and digitized at 10 kHz using a Digidata 1440A or 1550B digitizer (Molecular Devices, San Jose, CA, United States) and a PC computer equipped with commercial software (PCLAMP 10; Molecular Devices, San Jose, CA, United States) The standard sodium chloride bath solution contained (in mM): 150 NaCl, 10 CsCl, 4.7 KCl, 1 MgCl_2_, 1 CaCl_2_, 5 glucose, and 5 HEPES, with pH adjusted to 7.4 with NaOH. The cesium chloride pipette solution contained (in mM): 140 CsCl, 10 EGTA, 1 MgATP, 10 HEPES (pH 7.4) to which various amounts of calcium were added to obtain a final free [Ca^2+^]_*i*_ ranging between 200 and 600 nM, as calculated with the program EQCAL for Windows (BioSoft, Cambridge, United Kingdom). The *N*-methyl-*D*-glucamine (NMDG) chloride bath solution contained (in mM): 150 NMDG-Cl, 4.7 KCl, 1 MgCl_2_, 1 CaCl_2_, and 5 glucose, 5 HEPES (pH 7.4). The standard NMDG chloride pipette solution contained (in mM): 140 NMDG-Cl, 10 EGTA, 1 MgATP, and 10 HEPES (pH 7.4) to which various amounts of calcium were added to obtain a final, free calcium concentration ranging between 100 and 600 nM. All the experiments were conducted at room temperature (22–24°C).

### Statistical Analysis

Statistical analysis was performed with SigmaPlot 11 (Systat Software, San Jose, CA, United States) or Origin 2017 data analysis and graphing software (OriginLab, Northampton, MA, United States). The Student’s *t*-test was used to determine significant (*p* < 0.05) differences between means (as indicated by * symbols). Error bars in line and bar graphs indicate the mean ± standard error of the mean (SEM). The number of observations (n) is included to allow assessments of standard deviations (multiply the SEM by √n) and 95% confidence intervals (multiply the SEM by 1.96). Points in each bar graph indicate individual data points.

## Results

### Effects of Shear Stress

**FIGURE 1:**
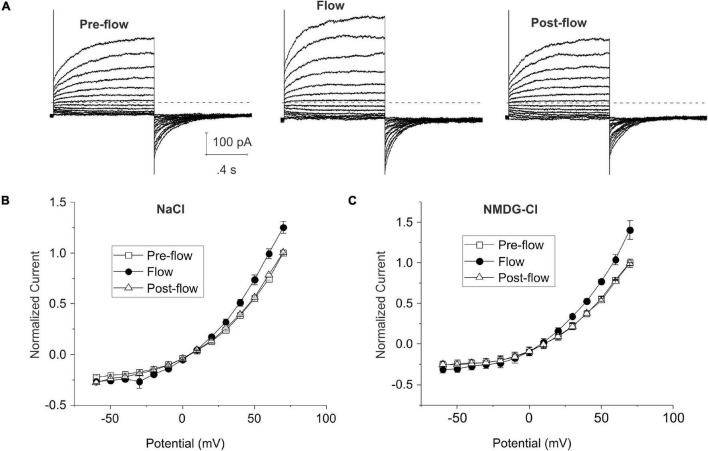
Shear stress induces changes in whole cell chloride current in differentiating lens cells. **(A)** Representative families of current traces recorded before, during and after fluid flow. Bath solution contained NaCl solution; patch pipette contained CsCl internal solution to which calcium was added to give a final free calcium concentration of ∼300 nM. The voltage clamp protocol consisted of a series of sequential steps from a holding potential of –60 mV to potentials ranging between –60 and 80 mV in 10 mV increments. The dashed line represents zero current level. **(B)** Effect of flow on the normalized steady-state I–V curve in standard NaCl solution (*n* = 4). The steady-state current measured at the end of the 20-s pulse was normalized to the steady-state current at +70 mV pre-flow and plotted as a function of voltage before (open squares), during flow (filled circles) and after cessation of flow (open triangles). **(C)** Effect of flow on the normalized steady-state I–V curve in NMDG-Cl solution (*n* = 6). Pipette solution consisted of NMDG-Cl containing 300 nM [Ca^2+^]_*i*_. The steady-state current measured at the end of the 20-s pulse was normalized to the steady-state current at +70 mV pre-flow and plotted as a function of voltage before (open squares), during flow (filled circles) and after cessation of flow (open triangles).

Figures 1A,B show representative families of current traces and mean steady-state I–V curves recorded from lens cells under standard whole cell conditions in the absence and presence of mechanical stress which was induced by focally perfusing the fiber cell at a constant rate with standard NaCl bath solution. CsCl internal solution containing ∼300 nM [Ca^2+^]_*i*_ was used in the pipette solution to activate endogenous calcium-activated chloride currents and block potassium currents in lens fiber cells (Tong et al., 2019). Under basal (pre-flow) conditions, most lens cells expressed a calcium-activated chloride current that slowly activated on depolarization. On repolarization to −60 mV, an inward tail current was observed that gradually deactivated over time. Fluid flow resulted in a significant increase in the size of the outward current (and corresponding inward tail current) evoked by depolarizing voltage clamp steps. The normalized steady-state outward current at 70 mV increased by 25 ± 6.0% (*n* = 4) (Figure 1B). The effects of fluid flow on the amplitude of the outward rectifying current could be reversed by stopping flow. Similar results were obtained when NMDG was used as the main cation in the bath and pipette solution to block current flow through non-selective cation channels indicating that the flow-induced increase in current was due to changes in an anion conductance (Figure 1C).

**FIGURE 2:**
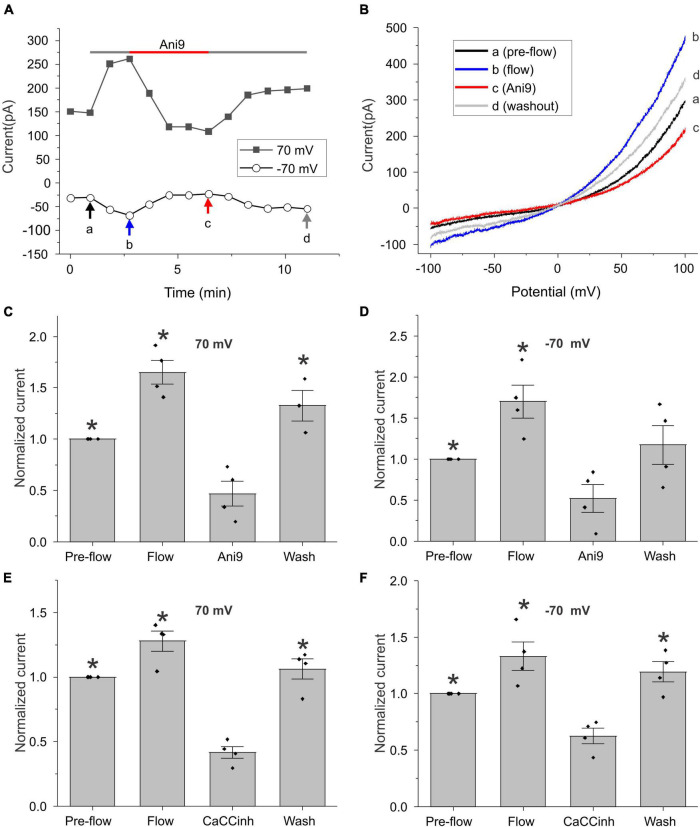
Effect of chloride channel blockers on the flow-induced current. **(A)** Representative experiment showing that fluid flow invoked an increase in current at ± 70 mV (measured during ± 100 mV voltage-clamp ramps) that could be blocked by 5 μM Ani9. Holding potential was –60 mV. Ramps from –100 to + 100 mV with a duration of 2 s were applied every 55 s. **(B)** Currents in response to ramps at points indicated by a, b, c, and d are shown in panel **(A)**. Bar graphs show the mean and standard error of the mean (SEM) (*n* = 4) for Ani9 and CaCCinh treatments. **(C,D)** The effect of 5 μM Ani9 on the amplitude of the flow-induced current at ± 70 mV normalized with respect to the value of the current at ± 70 mV pre-flow. Significant differences (*) are relative to Ani9 condition (*n* = 4). **(E,F)** The effect of 50 μM CaCCinh on the amplitude of the flow-induced current at ± 70 mV normalized with respect to the value of the current at ± 70 mV pre-flow. Significant differences (*) are relative to the CaCCinh condition (*n* = 4). In all cases, the bath solution consisted of standard NaCl external solution. Pipette solution consisted of CsCl internal solution containing 600 nM [Ca^2+^]_*i*_.

To further investigate this phenomenon, we monitored changes in the amplitude of the calcium-activated chloride current at ± 70 mV (measured during ± 100 mV voltage clamp ramps) as a function of time (Figure 2). As shown in Figure 2A for a representative cell, application of flow induced an increase in whole cell current that could be reversibly blocked by the TMEM16A specific CaCC blocker, Ani9 (Seo et al., 2016). Figure 2B shows the I–V relationship measured pre-flow (a), during flow (b), after application of Ani9 (c), and following washout of Ani9 (d), for the same cell as shown in Figure 2A. Both the pre-flow currents and the flow-induced currents displayed the characteristic outwardly rectifying shape of TMEM16A currents and had a reversal potential of approximately 0 mV. Following application of 5 μM Ani9, the current was reduced to a level below that of the pre-flow current suggesting that both components of the current were due to TMEM16A channels. The effects of Ani9 on the amplitude of the chloride current at +70 and −70 mV are summarized in Figures 2C,D. A similar reduction in the current was observed in response to the CaCC blocker, CaCCinh-A01 (De La Fuente et al., 2008; Namkung et al., 2011), as illustrated in Figures 2E,F.

**FIGURE 3:**
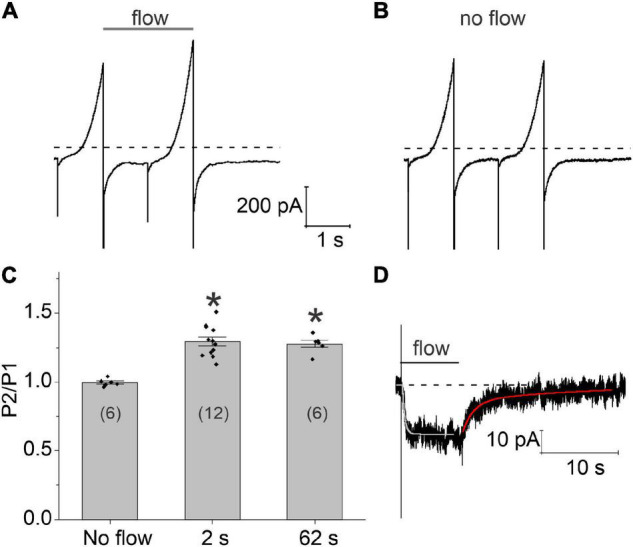
Time course of development and recovery of the flow-induced, chloride current. **(A)** Representative experiment showing the effect of fluid flow on the whole cell current using a paired ramp protocol. In this protocol, two 1 s ramps from –100 to + 100 mV were applied in succession from a holding potential of –60 mV. The time interval between the two ramps was 1 s. Application of fluid flow (gray bar) resulted in a significant increase in the amplitude of the current during the second (test) ramp pulse. **(B)** Whole cell currents recorded from the same cell in the absence of fluid flow. **(C)** Bar graph summarizing the effect of fluid flow on the amplitude of the membrane current peak at 100 mV recorded during the second ramp (P2) normalized with respect to the current peak at 100 mV recorded during the first ramp (P1). The number of cells tested is indicated within parentheses. Significant differences (*) are relative to the no flow condition. The bath solution consisted of NaCl external solution. The pipette consisted of CsCl internal solution containing 200–300 nM [Ca^2+^]_*i*_. **(D)** Representative experiment showing the effect of an 8 s fluid pulse on the holding current at –60 mV. Solid lines drawn through the data are single and double exponential fits to the rising (light gray) and declining (red) phase of the current, respectively. Bath solution consisted of NMDG-Cl external solution. Pipette solution consisted of NMDG-Cl internal solution containing ∼300 nM [Ca^2+^]_*i*_. Dashed line represents zero current level.

The time course of these flow-induced changes in chloride current were investigated by applying two 1 s ramps from −100 to + 100 mV in secession, separated by a variable time interval at a holding potential of −60 mV. A continuous stream of fluid was applied to the cell from the end of the first voltage clamp ramp to the end of the second ramp. Typical results are shown in Figure 3A. Application of a 2 s fluid pulse resulted in a significant increase in the amplitude of the current during the second (test) ramp. In contrast, no changes in the amplitude of the current were observed during the test ramp relative to the first ramp in the absence of flow (Figure 3B). Increasing the duration of the fluid pulse from 2 to 62 s failed to cause a further increase in the amplitude of the current during the test ramp suggesting that the flow-induced increase in chloride current had already reached steady-state by 2 s. The results of these experiments are summarized in Figure 3C.

In order to better define the time course of the flow-induced changes in chloride current, the cell was held at a constant holding potential of −60 mV while applying a fluid pulse of defined duration as illustrated in Figure 3D. When an 8 s fluid pulse was applied to the cell, the holding current rapidly increased to a new steady-state value. The time course of current increase could be described by fitting the holding current, following a short initial lag, to a single exponential with a mean time constant of 396.198 ± 74.5 ms (*n* = 5). Following cessation of flow, the holding current returned to its original baseline value. The time course of recovery could be described by the sum of two exponentials: a fast component with a time constant of 852.2 ± 217.2 ms and a slow component with a time constant of 11.26 ± 2.37 s (*n* = 5). The amplitude of the fast component relative to the slow component was 1.39 ± 0.27 (*n* = 5).

**FIGURE 4:**
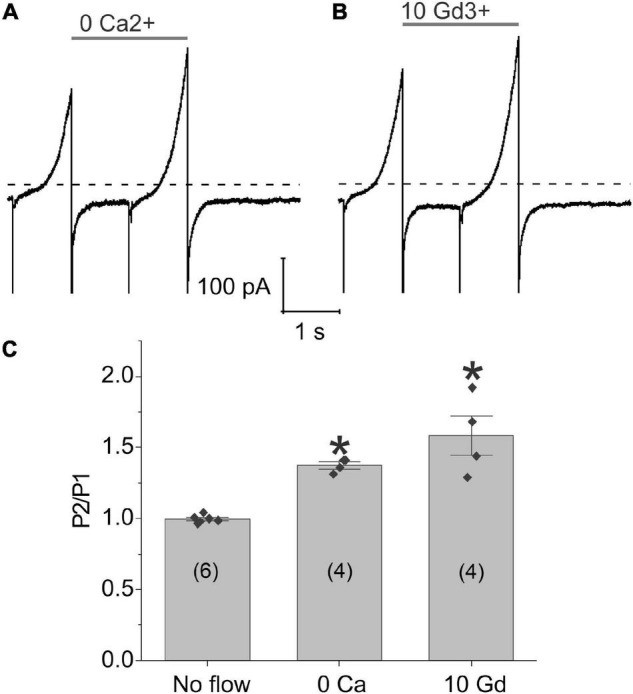
Application of shear stress caused a significant increase in chloride currents even in the absence of external calcium. Representative experiments showing the effect of fluid flow on the whole cell current recorded using a paired ramp protocol in panel **(A)** the nominal absence of external calcium or **(B)** the presence of 10 μM Gd^3+^. **(C)** Bar graph summarizing the effect of zero-added calcium and 10 μM Gd^3+^ on the amplitude of the membrane current recorded at 100 mV during the second ramp peak (P2) normalized with respect to the current recorded at 100 mV during the first ramp peak (P1) calculated as mean ± standard error of the mean (SEM). The number of cells tested is indicated within parentheses. Significant differences (*) are relative to the absence of flow. The bath solution contained NMDG-Cl solution. The pipette solution consisted of NMDG-Cl internal solution containing ∼300 nM [Ca^2+^]_*i*_. Dashed line represents zero current level.

To investigate the possible role of calcium influx through mechanosensitive channels in the generation of flow-induced calcium-activated chloride currents, we examined the effect of flow on the calcium-activated chloride current using a paired ramp protocol either in the nominal absence of external calcium or following application of gadolinium (Gd^3+^), a non-specific cation channel blocker which has been previously reported to block piezo channels (Coste et al., 2010), gap junctional hemichannels (Eskandari et al., 2002), L-type calcium channels (Biagi and Enyeart, 1990; Lacampagne et al., 1994), and some TRP channels (Yang and Sachs, 1989; Gunthorpe et al., 2002; Hamill, 2006). Removal of external calcium (Figure 4A) or application of 10 μM Gd^3+^ (Figure 4B) did not alter the response of the chloride current to fluid flow. To further test this hypothesis, we increased the concentration of Gd^3+^ to 100 μM Gd^3+^ and still saw no effect. The results of these experiments are summarized in Figure 4C. These findings suggest that calcium influx through mechanosensitive channels is not essential for the flow-induced response of the TMEM16A currents in lens cells.

### Interactions Between TRPV4 and Ca^2+^-Activated Chloride Currents

**FIGURE 5:**
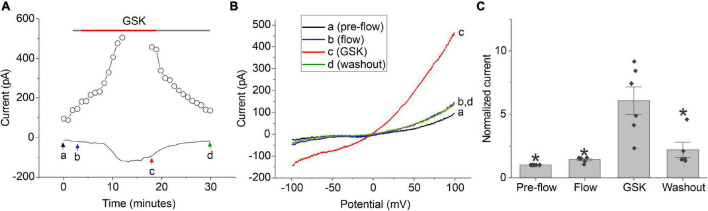
TRPV4 agonist-induced current response in dissociated lens cells. **(A)** Representative experiment showing the effect of 30 nM GSK (red bar) on the amplitude of the chloride current measured at + 100 mV (open circles) or –60 mV (solid line) in a lens cell. NMDG-Cl bath and pipette solutions were used to prevent contamination of the chloride current by non-selective cation currents. Holding potential was –60 mV. Ramps from –100 to + 100 mV with a duration of 2 s were applied every 55 s. **(B)** Currents measured in response to ramps at points indicated by a, b, c, and d in panel **(A)**. **(C)** Bar graph with individual data points (*n* = 6) indicating the effect of 30 nM GSK on the amplitude of the chloride current at +100 mV normalized to the value of the current at +100 mV measured pre-flow. Significant differences (*) are relative to the GSK condition.

Previous studies have shown that TRPV4 is expressed in the lens epithelial and peripheral fiber cells and can be activated by tension exerted by the ciliary zonules on the lens capsule (Chen et al., 2019). Thus, we hypothesized that another mechanism for regulating calcium-activated chloride currents in lens cells might involve calcium influx through TRPV4 channels. To test this hypothesis, whole cell patch clamp experiments were performed on dissociated lens cells using the selective TRPV4 agonist (GSK 1016790A; GSK) (Thorneloe et al., 2008) as illustrated in Figure 5. NMDG chloride bath and pipette solutions were used to prevent contamination of the chloride current by non-selective cation currents; the bath contained 1 mM calcium and the pipette solution contained 100 nM free calcium. To distinguish effect of GSK from the effect of shear stress, the cell was first perfused with control solution for 2–5 min prior to application of GSK. Upon activation of TRPV4 with 30 nM GSK, a robust increase in an outwardly rectifying, chloride current was observed whose I–V relationship became progressively more linear over time. Following washout of GSK, the membrane current partially returned to its original level prior to application of GSK. A similar increase in chloride current was observed in approximately half of the cells that were tested using this protocol. In the remainder of the cells, no response to GSK was observed.

**FIGURE 6:**
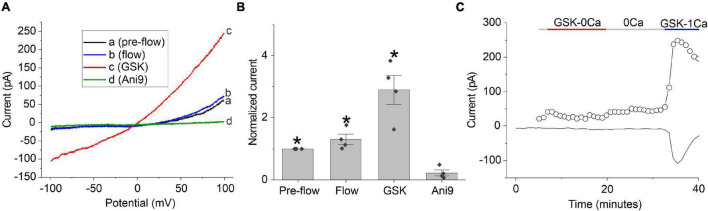
GSK-induced chloride currents can be blocked by application of 5 μM Ani9 or removal of external calcium. **(A)** Chloride currents evoked in response to ± 100 mV ramps of 2 s duration recorded pre-flow (black line), during flow (blue line), following application of GSK (red line) and after application of Ani9 (green line). **(B)** Bar graph summarizing the effect of 5 μM Ani9 on the amplitude of the GSK-induced current at + 100 mV normalized to the value of the current at + 100 mV measured pre-flow (*n* = 4). Significant differences (*) are relative to Ani9 treatment levels. **(C)** Representative experiment showing the effect of 30 nM GSK on the amplitude of calcium-activated chloride current measured at + 100 mV (open circles) and –60 mV (solid line) in NMDG-Cl bath solution, first in the absence of external calcium and then in the presence of 1 mM [Ca^2+^]_*o*_. A robust, GSK-stimulated chloride current was only observed in the presence of 1 mM [Ca^2+^]_*o*_. The pipette solution consisted of NMDG-Cl internal solution containing 100 nM [Ca^2+^]_*i*_.

The time- and voltage-dependent properties of chloride currents evoked by GSK resembled those of the calcium-activated chloride current, TMEM16A. To determine if TMEM16A contributes to the chloride currents evoked by GSK, the TMEM16A specific inhibitor, Ani9, was tested. The GSK-stimulated chloride currents were rapidly and completely inhibited following application of 5 μM Ani9 as shown in Figures 6A,B. To demonstrate that the effect of GSK on CaCCs was due to influx of calcium through TRPV4 channels, cells were first stimulated with the TRPV4 agonist, GSK, in the nominal absence of external calcium and then in presence of 1 mM [Ca^2+^]_*o*_. A representative experiment is shown in Figure 6C. The lens cell exhibited only small changes in chloride current in the absence of external calcium but developed robust, GSK-stimulated chloride current in the presence of 1 mM [Ca^2+^]_*o*_. Similar results were obtained in 2 other experiments.

## Discussion

The mechanisms of regulation and physiological functions of CaCCs in the lens are poorly understood. Here we show that mechanical stress created by focal perfusion resulted in an increase in an outwardly rectifying membrane current in differentiating lens epithelial cells and newly elongating fiber cells. This flow-induced current reversed polarity at the chloride equilibrium potential and could still be observed when NMDG-chloride was used as the main ion in the bath and pipette solution indicating that the flow-induced current was primarily carried by anions. In addition, both the flow-induced component and the basal component of the chloride current could be blocked by TMEM16A specific CaCC blocker, Ani9, suggesting that both components of the current were carried by TMEM16A chloride channels.

The kinetics of development and recovery of the calcium-activated chloride current from mechanical stress were very rapid, occurring in less than a second which closely parallels the time course of disaccommodation and accommodation previously described in humans and monkeys (Campbell and Westheimer, 1960; Croft et al., 1998). It is intriguing to speculate that these two processes might be correlated. The mechanism for chloride channel activation in response to fluid flow remains unclear. However, the lack of effect of removal of external calcium and the rapid kinetics of this response suggests that it might be due to tethering of the channel to the cytoskeleton. In support of this hypothesis, Perez-Cornejo et al. (2012) showed, using a proteomics approach, that TMEM16A associates with the signaling/scaffolding proteins ezrin, radixin, moesin, and RhoA, which link the plasma membrane to the actin cytoskeleton.

Chloride channels have been previously shown to be modulated by mechanical stress in other tissues. In vascular endothelial cells, perfusion activates a chloride current which causes endothelial depolarization. However, the molecular identity of these chloride channels is still not known and the flow-induced response had a slow time course of development suggesting that it was unlikely to be due to a direct action of membrane or cytoskeletal strain on the chloride channel (Olesen et al., 1988; Barakat et al., 1999; Gautam et al., 2006). More recently, shear stress has been shown to stimulate TMEM16A chloride channels in biliary epithelial cells indicating that these channels were mechanosensitive (Dutta et al., 2011, 2013). However, once again the time course of this response was much slower than that observed in lens cells and depended on ATP and P2Y receptors.

Another potential mechanism for activation of CaCCs in the lens involves an increase in cytosolic calcium due to calcium-influx through transient receptor potential cation channel, subfamily V member 4 (TRPV4) channels which are thought to act as mechanosensors in the lens and modulate lens intracellular hydrostatic pressure (Shahidullah et al., 2012a,b, 2015; Gao et al., 2015; Chen et al., 2019; Delamere et al., 2020). Our results show that pharmacological activation of TRPV4 by GSK results in a marked enhancement of the calcium-activated chloride current at both positive and negative potentials in fiber cells isolated from DblKO mice that lack Cx50 and Cx46. Removal of calcium from the extracellular solution blocked this response suggesting that it was due to calcium-influx through TRPV4 channels. In lenses from wild-type mice, the response to GSK is likely to also be reinforced by release on calcium from internal stores by a process involving the opening of Cx50 hemichannels (Delamere et al., 2020). Similar pathways involving coupling between TMEM16A and TRPV4 have been shown to stimulate TMEM16A channels in other tissues such as the choroid plexus, salivary glands and lacrimal glands and contribute to fluid secretion (Takayama et al., 2014; Derouiche et al., 2018).

Previous immunohistochemical studies have shown that TMEM16A and B are expressed in the zone of fluid efflux of lens (Tong et al., 2019) along with aquaporins (Petrova et al., 2018, 2020), TRPV4 (Nakazawa et al., 2019), and K^+^-Cl^–^ cotransporters (Chee et al., 2006) suggesting that CaCCs might be involved in the regulation of fluid efflux from the lens in response to mechanical stress caused by an increase in zonular tension. In this scheme, direct mechanical strain and local entry of calcium through TRPV4 channels would activate nearby CaCCs in the peripheral most lens cells and result in chloride efflux due to the outwardly directed electrochemical gradient for chloride in these cells. This chloride efflux and accompanying potassium efflux would in turn osmotically drive water efflux through aquaporins. In contrast, in the deeper cell layers where the driving force for chloride is inward, activation of CaCCs would result in chloride influx and fluid gain. Since all the cells in the lens are connected by gap junctions which allow the flow of ions and water, this would be expected to cause the redistribution of free water throughout the lens and might contribute to the changes in changes in lens volume and water content that occur in response to changes in surface pressure or zonular tension. In addition, changes in the water content of the lens would be expected to alter the gradient of refractive index (GRIN) and change the optical properties of the lens (Donaldson et al., 2017). Over the long term, modulation of the activity of CaCCs could also direct phenotypic changes, such as fiber cell elongation or epithelial cell proliferation. These changes could contribute to the global alterations in eye function observed in myopia and presbyopia. Further studies are required to better understand the physiological and pathophysiological role of mechanosensitivity of calcium-activated-chloride-channels.

## Data Availability Statement

The original contributions presented in the study are included in the article/supplementary material, further inquiries can be directed to the corresponding author.

## Ethics Statement

The animal study was reviewed and approved by the Rosalind Franklin University Animal Care and Use Committee.

## Author Contributions

PA and J-JT performed the electrophysiology experiments. LE performed and supervised the electrophysiology studies, analyzed the functional data, and provided the overall oversight for the design and execution of the work. All authors contributed to the preparation of the manuscript.

## Conflict of Interest

The authors declare that the research was conducted in the absence of any commercial or financial relationships that could be construed as a potential conflict of interest.

## Publisher’s Note

All claims expressed in this article are solely those of the authors and do not necessarily represent those of their affiliated organizations, or those of the publisher, the editors and the reviewers. Any product that may be evaluated in this article, or claim that may be made by its manufacturer, is not guaranteed or endorsed by the publisher.
